# Yy1 regulates Senp1 contributing to AMPA receptor GluR1 expression following neuronal depolarization

**DOI:** 10.1186/s12929-019-0582-1

**Published:** 2019-10-20

**Authors:** Tao Wu, Mary E. Donohoe

**Affiliations:** 1 School of Basic Medicine and Clinical Pharmacy, China Pharmaceutical University, Nanjing, Jiangsu 210009 People’s Republic of China; 2 Burke Medical Research Institute, White Plains, NY 10605 USA; 3 Department of Neuroscience, Brain Mind Research Institute, Department of Cell & Development, Weill Cornell Medical College, New York, NY 10065 USA; 4 Present address: Department of Medicine, Division of Regenerative Medicine, University of California San Diego School of Medicine, La Jolla, CA 92037 USA

**Keywords:** Senp1, Neuronal membrane depolarization, Yy1, Brd4, Phosphorylation, GluR1

## Abstract

**Background:**

Neuronal activity-induced changes in gene expression patterns are important mediators of neuronal plasticity. Many neuronal genes can be activated or inactivated in response to neuronal depolarization. Mechanisms that activate gene transcription are well established, but activity-dependent mechanisms that silence transcription are less understood. It is also not clear what is the significance of inhibiting these genes during neuronal activity.

**Methods:**

Quantitative Real Time-PCR, western blot and immunofluorescence staining were performed to examine the expression of Senp1 and GluR1 in mouse cortical neurons. The alterations of Yy1 phosphorylation upon neuronal depolarization and the interaction of Yy1 with Brd4 were studied by protein co-immunoprecipitation. The regulators of Yy1 phosphorylation were identified by phosphatase inhibitors. Chromatin immunoprecipitation, in vitro DNA binding assay, luciferase assay and gene knockdown experiments were used to validate the roles of Yy1 and its phosphorylation as well as Brd4 in regulating Senp1 expression.

**Results:**

We report that neuronal depolarization deactivates the transcription of the SUMO protease *Senp1*, an important component regulating synaptic transmission, scaling, and plasticity, through Yy1. In un-stimulated neurons, *Senp1* transcription is activated by a Yy1-Brd4 transcription factor protein complex assembled on the *Senp1* promoter. Upon membrane depolarization, however, Yy1 is dephosphorylated and the Yy1-Brd4 complex is evicted from the *Senp1* promoter, reducing *Senp1* transcription levels. Both Yy1 and Senp1 promote the expression of AMPA receptor subunit GluR1, a pivotal component in learning and memory.

**Conclusions:**

These results reveal an axis of Yy1/Brd4-Senp1 which regulates the expression of GluR1 during neuronal depolarization. This implicates a regulation mechanism in silencing gene expression upon neuronal activity.

## Background

Neuronal plasticity is a key property of neurons for short- or long-lasting phenotypes in response to different external stimuli and cellular scenarios and is fundamental for normal brain developmental functions such as learning and memory [1, 2]. The importance of plasticity is underscored in pathologies such as autism spectrum disorders and neurodegenerative diseases [1]. AMPA receptors (AMPARs) mediate the fast-excitatory synaptic transmission in the mammalian central nervous system and are pivotal for long-lasting memory, dynamic changes in neuronal synaptic efficiency or plasticity, and synaptic strength underlying plasticity [3–7]. The AMPAR subunit, GluR1, is crucial for learning and memory [8]. Neuronal activity can regulate AMPAR trafficking to change its synaptic localization transiently, while alteration of the total amount of the protein by the activity could be more important for long-term modulation of synaptic efficacy [9–13]. However, it is still not clear about the pathways that alter the total expression level of AMPAR upon neuronal activity.

In response to membrane depolarization, neurons rapidly deploy protein posttranslational modifications (PTMs), such as phosphorylation, that modify neural protein activities and signaling transduction. Membrane depolarization modulates the activities of many protein kinases and phosphatases to maintain a neuronal response to environmental stimuli [14, 15]. The role for other PTMs (such as acetylation, ubiquitination, or SUMOylation) during neuronal activity is not clear. SUMOylation is the covalent conjugation of the small protein SUMO (Small Ubiquitin like Modifier) to its protein substrates. SUMOylation is a dynamic process involving a cascade of enzymes [16, 17]. The E3 ligases and Sentrin/SUMO-specific proteases (Senps) specifically regulate the SUMOylation/deSUMOylation of protein targets, respectively [18]. Protein SUMOylation is regulated by neuronal activity and participates in the synaptic transmission, homeostatic synaptic scaling, and plasticity [19, 20]. SUMOylation is required for glycine-induced increase in AMPAR on hippocampal neurons. Senp1 is one of the major proteases that can remove the SUMO covalent modification from target proteins. Over-expression of Senp1 or inhibition of SUMOylation decreases AMPAR surface expression [21], implicating SUMOylation as an important regulator of AMPAR trafficking during plasticity. As the major regulators of protein SUMOylation, Senps may play key roles in this process [20, 22], however, it is not defined how neuronal activity regulates SUMO dynamics and Senps’ activity.

Neurons maintain precise receptor-mediated excitability versus inhibitory signals by balancing the activation and repression of gene transcription [23]. Several transcription factors, such as c-fos, and Egr 1/2 are well-established activators of gene expression in response to neuronal activity [24]. In general, the mechanisms that activate gene transcription in response to neuronal depolarization have received substantial attention, but recent transcriptome studies in mouse primary neurons have revealed that neuronal depolarization can repress the transcription of approximately an equal number of genes [25]. The mechanisms that mediate activity-dependent repression, however, are not well understood even though accumulating evidence suggests that these mechanisms play a crucial role in normal brain function [26, 27].

Here, we show that membrane depolarization deactivates the transcription of the SUMO protease Senp1 in primary cortical neurons. In un-stimulated neurons, we demonstrate that the multifunctional transcription factor Yy1 recruits the bromodomain protein Brd4 to the *Senp1* promoter, where the Yy1-Brd4 activates *Senp1* transcription. Upon membrane depolarization, Yy1 is dephosphorylated by the protein phosphatase PP1/PP2A and this leads to the eviction of both Yy1 and Brd4 from the *Senp1* promoter. In addition, we show that Yy1-Senp1 axis drives the expression of GluR1 in unstimulated neurons. Overall, our studies reveal a molecular mechanism for neurons to dampen gene expression upon neuronal membrane depolarization, which could be applied to neuronal plasticity.

## Methods

### Cells, reagents, and antibodies

Human embryonic kidney (HEK) 293 T and Neuro2A cells were cultured as described [28]. The mouse Yy1 expression vectors were engineered by PCR cloning into pCMV5-Flag vector or CMV-Myc vector (Clontech). To clone the promoter of *Senp1*, 2541-base pair (bp) upstream of the transcription start site (TSS) of *Senp1* was amplified from mouse genomic DNA and inserted into pGL3-basic vector (Promega) with SacI/BglII. The Yy1-S184, 247A mutant and wild type genes were subcloned into a CMV-Myc expression vector using previously described Yy1 mutant and Yy1-wild type vectors [29] (gifts from Dr. Patrizia Casaccia) as PCR templates. The full-length Brd4 was generated using pcDNA4cBrd4 (AddGene #14441) as a PCR template and cloned into a Myc-tag containing vector. The N-terminus of Brd4 containing the two bromodomains was amplified by PCR cloned into the CMV Myc epitope-tagged vector.

The short interfering RNAs (siRNAs) against mouse *Yy1* and Brd4 (SASI_Mm01_00116324) were purchased from Sigma and transfected into cells using Lipofectamine RNAiMAX (Invitrogen) following the manufacture’s instructions. Yy1 shRNA constructs were cloned into pSilencer-EGFP vector (gift from Dr. Tao Sun) with *XhoI/EcoRI.* The following sequences were used for shRNA vectors: shYy1–1: 5’ACATCTTAACACACGCTAAAGCTTCAAGAGAGCTTTAGCGTGTGTTAAGATGTTTTTTT3’; shYy1–2: 5’GCCTCTCCTTTGTATATTATTAAGTTCTCTAATAATATACAAAGGAGAGGCTTTTTT3’; and shYy1–3: 5’ACAGAAAGGGCAACAATAATTCAAGAGATTATTGTTGCCCTTTCTGTTTTTTT3’. All the constructs were confirmed by sequencing. The following antibodies were used for western blot and/or chromatin immunoprecipitation: anti-Flag M2 beads (Sigma-Aldrich), anti-Histone 4 acetyl (H4Ac) (Active Motif), anti-Myc (Sigma-Aldrich), anti-Flag (Sigma-Aldrich), anti-IgG (Santa Cruz), anti-Brd4 (Bethyl), anti-Yy1 (Santa Cruz), anti-phospho-Serine (Abcam), and anti-Senp1 (Santa Cruz), anti-GluR1 (Millipore, ABN241).

### Luciferase reporter assays

Luciferase reporter constructs containing *Senp1* promoter and the wild type or mutated Yy1 expression constructs were co-transfected into Neuro2a cells using Lipofectamine 2000 (Invitrogen). The pRL-TK vector (Promega) was used as an internal transfection control. Cells were lysed 48 hours(hrs) post-transfection and subjected to the Dual Luciferase Reporter Assay (Promega). Relative luciferase activity was obtained by dividing the firefly luciferase activity (from the luciferase reporter constructs) by the *Renilla* luciferase activity (from pRL-TK vector). All experiments were performed in triplicate. Error bars represent one standard deviation from the mean.

### Primary mouse cortical cultures, membrane depolarization, chemical treatment and knockdown in cortical neurons

Mouse primary neurons were isolated from E16.5 C57BL/6 mouse embryo cortices as described [20]. For membrane depolarization, neurons cultured for 7 days in vitro (DIV7) were incubated for either 2 hrs or 24 hrs in media with or without different doses of KCl (25 mM or 60 mM KCl). For the chemical treatment in cortical neurons, 2 μM JQ1 (BPS Biosciences) [28], 50 μg/ml cyclosporin (CsA) (Santa Cruz), and 100 nM Okadaic acid (Santa Cruz) were dissolved in DMSO (vehicle) and added into the culture media. To perform Yy1 knockdown in cortical neurons, siRNAs were transfected into neurons with Lipofectamine RNAiMAX (Invitrogen). To deplete Yy1 using shRNAs, the shRNA plasmids were suspended in PBS and introduced into neurons by nucleofection using the Amaxa Nucleofector II.

### RNA extraction and quantitative RT-PCR

Total RNA was extracted from the cells using TRIzol reagent (Invitrogen Corporation, Carlsbad, CA), DNase I treated, and precipitated with isopropanol overnight. The cDNA was synthesized using the SuperScript III First Strand Synthesis System (Invitrogen). Quantitative reverse-transcribed PCR reactions were analyzed using SYBR Green PCR master mix (Applied Biosystems). All experiments were performed in triplicate and normalized with the housekeeping gene beta-actin. Statistical analyses (*p* values) were obtained using two-tailed unpaired *t* test. RT-PCR primers are available upon request.

### DNA binding assay

An in vitro DNA binding assay was performed as previously described with modifications [30]. Briefly, Myc-Yy1 wild type and Myc-Yy1-S184, 247A expression constructs were transfected into Neuro2A cells, immunoprecipitated with anti-Myc antibodies, and immobilized onto Protien A/G Plus-Agarose beads (Santa Cruz). These beads were then resuspended in a DNA binding buffer (20 mM Tris-Cl, pH 8.0, 75 mM KCl, 10 mM MgCl_2_, 10μM ZnCl_2_, 5% glycerol). *Senp1* promoter harboring 2511-bp upstream from the mouse *Senp1* TSS was amplified by PCR and purified with the QIAquick gel extraction kit (Qiagen). Approximately 4 μg of purified *Senp1* promoter DNA was incubated with the beads immobilized with Yy1 proteins at room temperature for 1.5 h. The beads were first washed with DNA binding buffer containing 350 mM NaCl twice, then washed with DNA binding buffer containing 150 mM NaCl twice, and finally washed with DNA binding buffer three times. Bound DNA was eluted with 100 mM NaHCO_3_ and 1% SDS. The proteins were digested with proteinase K. DNA was purified with phenol/chloroform and subjected to quantitative Real-time PCR using primers spanning  80-bp upstream of the *Senp1* TSS.

### GluR1 Immunostaining

Transfected cortical neurons were fixed with 4% formaldehyde for 15 min, blocked with 3% skim milk for 2 hrs and incubated with GluR1 antibody (1:100) at 4 °C. After overnight incubation, cells were washed with PBST and incubated with Alexa Fluor 555 goat anti-rabbit IgG (Invitrogen). Images were taken with a Nikon ECLIPSE 80i. GFP positive cells were selected to quantify their GluR1 signals with ImageJ software. Two-tailed unpaired student *t* test was used for the statistical analysis.

To stain surface GluR1, we incubated cultured neurons with GluR1 antibody (Millipore, ABN241) (1:100) in medium and kept them in the incubator for 1 hr. This antibody recognizes surface GluR1 extracellular N-terminal domain. Excess antibodies were then washed away with PBS. After fixation, cells were blocked followed by Alexa Fluor 555 goat anti-rabbit IgG (Invitrogen) secondary staining. Images were captured using the Nikon ECLIPSE 80i. GFP positive cells were selected to quantify their surface GluR1 signals with ImageJ. Two-tailed unpaired student *t* test was used for the statistical analysis.

### Protein co-immunoprecipitation (CoIP) and western blot

To detect endogenous Yy1 phosphorylation and Brd4-Yy1 interaction, adult mouse brain or mouse primary cortical neurons with or without KCl treatment were harvested. Proteins were extracted with CoIP buffer and first pre-cleared with agarose beads for 2 hrs at 4 °C. Six μg of anti-Yy1 antibodies or Brd4 antibodies were added to immunoprecipitate endogenous Yy1 or Brd4 overnight. Agarose beads (Santa Cruz) were then added and rotated for 4 hrs at 4 °C. After washed with CoIP buffer for 5 times, proteins were eluted and boiled in SDS-PAGE loading buffer and subjected to western blot analysis.

### Quantitative chromatin immunoprecipitation (qChIP)

Mouse cortical neurons with or without KCl depolarization were crosslinked with 1% formaldehyde for 10 min and processed for qChIP essentially as described [28]. The chromatin was fragmented to approximately 200–1000-bp by sonication. The sheared chromatin was first pre-cleared for 2 hrs rocking at 4 °C with agarose beads (Santa Cruz). Then 3 μg of the designated antibodies were added to immunoprecipitate the protein-DNA complex overnight at 4 °C. All the primers used for quantitative ChIP are available upon request.

## Results

### *Senp1* expression is down regulated in response to membrane depolarization

**Fig. 1:**
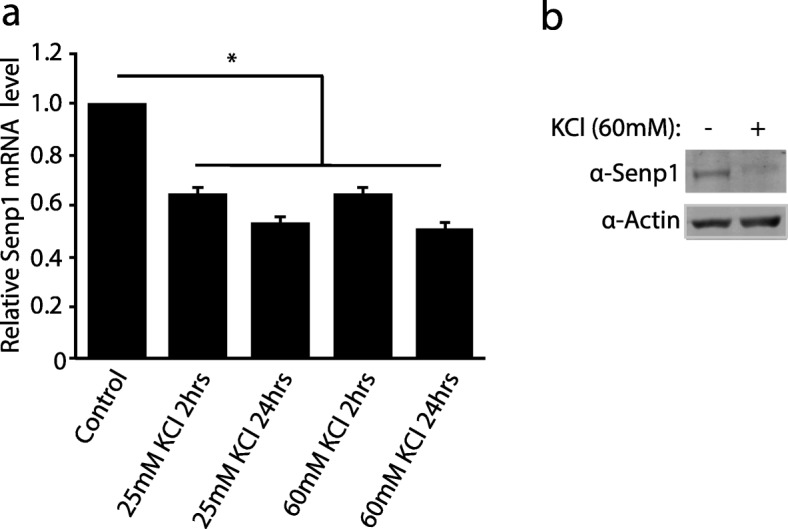
Membrane depolarization represses the SUMO protease *Senp1.* (**a**) The relative mRNA levels of *Senp1* in cortical neurons was determined by qRT-PCR following treatment with vehicle (Control), 25 mM potassium chloride (KCl) for 2 hours (hrs), 25 mM KCl for 24 hrs, 60 mM KCl for 2 hrs, or 60 mM KCl for 24 hrs. Graphs indicate three independent biological replicates. Error bars represent one standard deviation from the mean. *(*p* < 0.05). *p* value was determined using two-tailed unpaired *t* test. (**b**) Western blot analysis of Senp1 expression level following neuronal depolarization in cortical neurons. Total proteins were extracted from cortical neurons after 2 hrs treatment with 60 mM KCl and control treatment. Actin was used as loading control.

To better understand the molecular mechanisms that mediate transcriptional repression in response to membrane depolarization in neurons, we evaluated the expression levels of several chromatin and epigenetic modulators in mouse primary cortical neurons depolarized with potassium chloride (KCl). In this model system, KCl-mediated depolarization leads to an influx of calcium through the L-type voltage-sensitive channels that potentiates changes in gene expression patterns [24]. KCl-depolarized neurons increase the expression of *Bdnf* and CREB phosphorylation, which is consistent with other studies (Additional file 1: Figure S1a and S1b). By comparing their relative mRNA levels of KCl-stimulated neurons versus un-stimulated neurons, we found that only the *Senp1* and *Tet1* genes were significantly reduced by membrane depolarization among several tested genes (Additional file 1: Figure S1c and S1d). In addition, using two different doses of KCl (25 mM and 60 mM) at different times (2 hrs and 24 hrs), we found that both treatments reduced the expression of *Senp1* (Fig. 1a). Western blot analysis confirmed the silencing of Senp1 at the protein level following KCl treatment (Fig. 1b). In contrast, TTX treatment increased the expression of Senp1 **(**Additional file 1: Figure S1e). Recent work has shown that *Tet1*, an enzyme that converts the repressive 5′-methyl cytosine to the active 5′-hydroxymethylation DNA modification, is repressed after neuronal stimulation and/or fear conditioning [26], consistent with our results. In the current study, however, we have focused only on *Senp1* expression. Taken together, these results show that the expression of SUMO-specific protease gene *Senp1* is regulated by neuronal activity, with membrane depolarization reducing its expression.

### Yy1 activates *Senp1* transcription

**Fig. 2:**
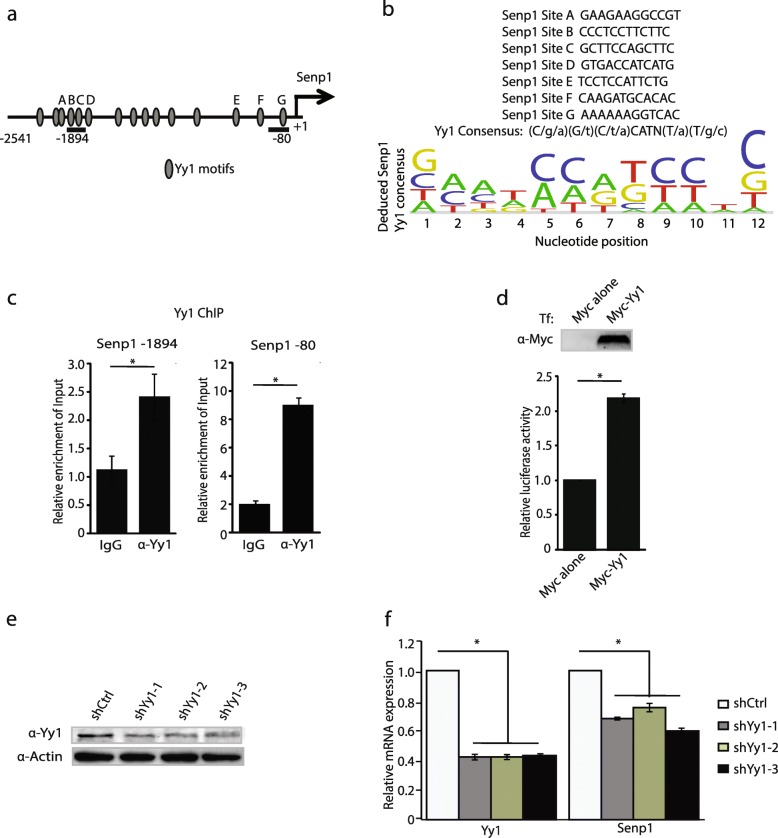
Yy1 binds to *Senp1* promoter and activates its transcription in unpolarized neurons. (**a**) Scheme showing the 2.5-kilobases (kb) region upstream of the transcription start site (TSS) (designated by + 1) of mouse *Senp1*. In silico analysis shows numerous Yy1 binding motifs are present on *Senp1* promoter. (**b**) Deduced Yy1 consensus at *Senp1*. (**c**) Yy1 is enriched on *Senp1* promoter. Chromatin was harvested from mouse cortical neurons and qChIP was performed with primers sets − 1894 and − 80. Relative enrichment was normalized with 2% input. Graphs indicate three independent biological replicates. Error bars represent one standard deviation from the mean. Two different PCR amplicons situated 1894 bp and 80 bp upstream of the *Senp1* TSS (− 1894 and − 80) were used for quantitative chromatin immunoprecipitation (qChIP). The PCR − 1894 amplicon spans the region of − 1894 to − 1741, whereas, the PCR − 80 amplicon spans the region between − 80 to + 7 relative to the *Senp1* TSS. (**d**) Yy1 activates *Senp1* promoter in luciferase assay. Upper panel verifies that Myc-Yy1 is over expressed in the cells subjected to the luciferase assay by immunoblot using anti-Myc antibodies. Lower panel shows that Yy1 transactivates the *Senp1* promoter using a standard luciferase reporter assay. Luciferase activity was measured in Neuro2A cells co-transfected with luciferase reporter containing *Senp1* promoter co-transfected with Myc-Yy1 or Myc alone (empty vector). Graphs indicate three independent biological replicates. Error bars represent one standard deviation from the mean. (**e**) Knockdown of Yy1 in Neuro2A cells by short hairpin RNAs (shRNAs). Whole cell extracts were prepared 46-48 hrs post transfection and immunoblotted with anti-Yy1 and anti-Actin antibodies. (**f**) Knock down of Yy1 reduces the expression of *Senp1*. Graphs indicate three independent biological replicates. Error bars represent one standard deviation from the mean. *(*p* < 0.05). *p* value was determined using two-tailed unpaired *t* test

To further study the underlying mechanisms, we first performed an in silico analysis of the Senp1 promoter using Jasper (http://jaspar.binf.ku.dk/) to identify potential DNA-specific binding transcriptional regulators. Our search results revealed numerous Yy1 binding sites within a 2.5-kilobase (kb) region upstream of the *Senp1* transcriptional start site (TSS) (Fig. 2a). We deduced a consensus Yy1 motif for the *Senp1* region (Fig. 2b). To determine whether Yy1 associates with the promoter region of *Senp1* in neurons, quantitative chromatin immunoprecipitation (qChIP) was performed on chromatin prepared from adult whole mouse brain at two different *Senp1* amplicons within the promoter region (situated at 80- and 1894-basepair (bp) upstream of the TSS, respectively) (Fig. 2a). Our results reveal Yy1 is enriched at the *Senp1* promoter in neurons as compared with control at both the − 1894 and − 80 regions (Fig. 2c).

Yy1 can function as either a transcription repressor or activator [31]. To determine its transcription regulatory function on the *Senp1* promoter, we co-transfected resting neurons with full-length myc-tagged Yy1 expression constructs and a plasmid harboring a 2541-bp fragment upstream of the mouse *Senp1* TSS region driving luciferase expression. The co-expression of Yy1 stimulated luciferase activity as compared to control (Fig. 2d), indicating that Yy1 is a transcriptional activator for the *Senp1* promoter. To establish that Yy1 is necessary for *Senp1* transcription, we constructed short hairpin Yy1 RNAs (shRNAs) targeting three different regions within Yy1 and transfected these vectors into Neuro2A cells. As shown in Fig. 2e, shYy1–1, shYy1–2, and shYy1–3 deplete the Yy1 protein. The knockdown of Yy1 compromises the levels of *Senp1* mRNA (Fig. 2f). Taken together, these results show that Yy1 activates Senp1 transcription in non-depolarized cells by directly binding the *Senp1* promoter.

### Yy1 forms a complex with Brd4 on the *Senp1* promoter

**Fig. 3:**
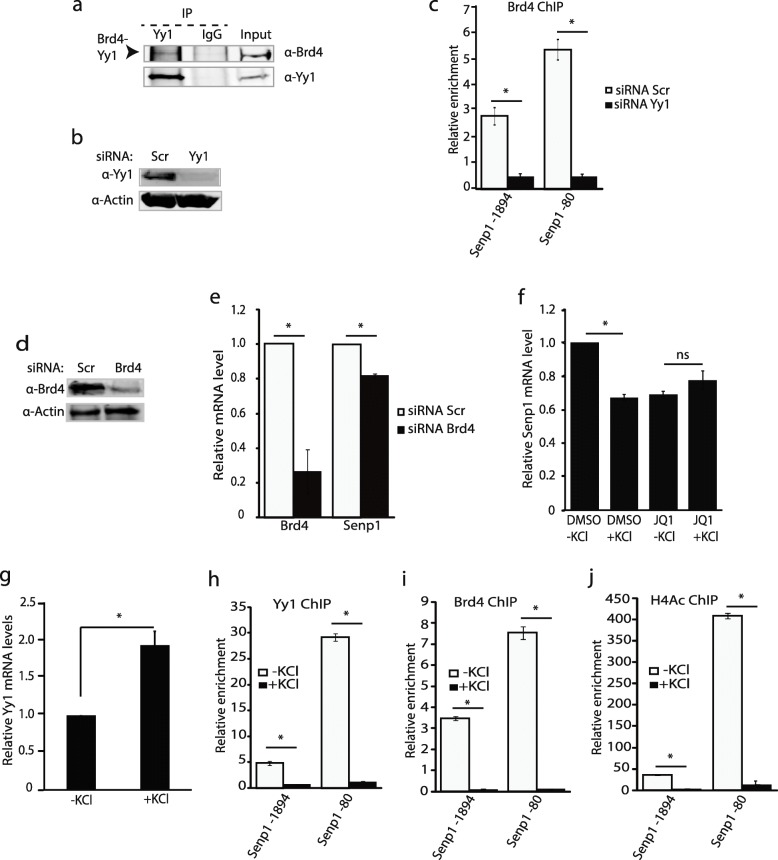
Neuronal depolarization evicts Yy1 and its interacting partner, the BET family member Brd4, from the *Senp1* promoter. (**a**) Yy1 partners with Brd4 in cells. Co-immunoprecipitation of Yy1 and Brd4 in Neuro2A cells. Arrowhead indicates the Brd4 pulled down by Yy1. (**b**) Depletion of Yy1 using short interfering RNAs (siRNAs). Neuro2A cells were transfected with Scramble (scr) or siYy1. Knockdown was confirmed by immunoblotting whole cell extracts with anti-Yy1 and anti-Actin antibodies. (**c**) Yy1 recruits Brd4 to the Senp1 promoter. qChIP of Brd4 at − 1894 and − 80 following Yy1 siRNA. (**d**) Brd4 knockdown in Neuro2A cells. Western blot of Brd4 and scramble (Scr) control following knockdown in Neuro2A cells. Actin is used as a protein loading control. (**e**) Knockdown of *Brd4* represses *Senp1* transcription. (**f**) BET inhibition by JQ1 induces alterations of *Senp1* mRNA levels. Cortical neurons were pretreated with 2 μM JQ1 for 22 hrs followed by 2 hr treatment with 60 mM KCl. The level of *Senp1* relative mRNA levels were quantitatively analyzed by qRT-PCR. (**g**) The relative *Yy1* mRNA levels after KCl treatment. Following 2 h KCl exposure, *Yy1* mRNA levels increase. (**h**) The binding of Yy1 on the *Senp1* promoter is inhibited by the neuronal depolarization. Chromatin was harvested from mouse cortical neurons with or without neuronal depolarization with 60 mM KCl for 2 hrs. The binding of Yy1 and IgG at *Senp1 promoter* was analyzed by qChIP with primers sets − 1894 and − 80. Relative enrichment was normalized with IgG. (**i**) Neuronal depolarization abolishes Brd4’s binding on the *Senp1* promoter. Chromatin was harvested from unstimulated or stimulated mouse cortical neurons with 60 mM KCl for 2 hrs. Brd4 antibodies were qChIP with primers sets − 1894 and − 80. Relative enrichment was normalized with IgG. (**j**) Histone H4 acetylation (H4Ac) levels are also depleted at the Senp1 promoter following depolarization. The binding of H4Ac, and IgG at *Senp1 promoter* was analyzed by qChIP using PCR primers sets corresponding to − 1894 and − 80 *Senp1* TSS. Relative enrichment was normalized with IgG. Graphs indicate three independent biological replicates. Error bars represent one standard deviation from the mean. *(*p* < 0.05). *p* value was determined using two-tailed unpaired *t* test

The protein interaction partners of Yy1 are important determinants of its transcriptional activity [31]. A recent report showed that Yy1 interacts with Brd4 [32], a member of the BET family that contain two bromodomains and recognizes acetylated histones. Brd4 recruits Positive Elongation Factor, pTEF-b, phosphorylating RNA polymerase II (RNAP Pol II) Serine 2, which results in a release from gene pausing and facilitates productive gene transcriptional elongation [33, 34]. RNAP Pol II pausing is essential for the rapid induction of immediate early genes in response to stimuli in neurons [35]. To address whether Brd4 also participates in the depolarization-induced *Senp1* downregulation, we first confirmed the physical interaction between Yy1 and Brd4 by co-immunoprecipitation (Co-IP) in neurons (Fig. 3a). With qChIP, we then established that Brd4 occupancy at the *Senp1* promoter was altered following Yy1 knockdown (Fig. 3b, c). Consistent with a role for Brd4 in activating gene transcription, we found that *Senp1* expression was compromised following Brd4 knockdown (Fig. 3d, e). When neuronal Brd4 was inhibited by the small molecule JQ1 which prohibits the binding of Brd4 bromodomain to acetylated histones [28, 36], KCl treatment did not further reduce the *Senp1* levels suggesting that the depletion of Brd4 could be a major factor for neuronal depolarization induced Senp1 repression (Fig. 3f). Collectively our results demonstrate that Yy1 targets the BET family member Brd4 to the *Senp1* promoter activating its transcription.

### Membrane depolarization evicts the Yy1/Brd4 complex from the *Senp1* promoter

To determine the role for Yy1 in *Senp1* repression during neuronal depolarization, we asked whether membrane depolarization could directly alter *Yy1* transcription. *Yy1* mRNA levels increase 2 hour following depolarization (Fig. 3g). In contrast, we did not observe a change in the Yy1 protein levels after 2 hr of KCl treatment (data not shown). To resolve the paradox between transcriptional up-regulation of *Yy1* and *Senp1* down-regulation upon neuronal activity, we examined Yy1 occupancy on the *Senp1* promoter in vivo using qChIP after membrane depolarization of primary mouse cortical neurons. We interrogated two different regions (− 1894 and − 80) upstream of the *Senp1* TSS for Yy1 occupancy. When compared to the un-stimulated neurons, the enrichment of Yy1 on both *Senp1* chromatin sites is greatly depleted after depolarization (Fig. 3h).

Because Yy1 recruits Brd4 to the *Senp1* promoter, we examined whether membrane depolarization might also affect Brd4’s occupancy at these regions. qChIP was performed using Brd4 antibodies on chromatin prepared from un-stimulated and depolarized neurons. Our qChIP analysis showed that the in vivo binding of Brd4 to the *Senp1* chromatin was depleted in response to neuronal activity (Fig. 3i). We also observed a dramatic reduction of Histone 4 acetylation (H4Ac) on these regions after neuronal depolarization (Fig. 3j). Our findings agree with previous studies suggesting that histone acetylation is dynamic and regulated by neuronal activity [37]. Taken together, we show that both Yy1 and Brd4 proteins are depleted from the *Senp1* promoter upon membrane depolarization, and the histone PTM, H4 acetylation is reduced.

### Membrane depolarization decreases Yy1 phosphorylation status resulting in its removal from the *Senp1* promoter

**Fig. 4:**
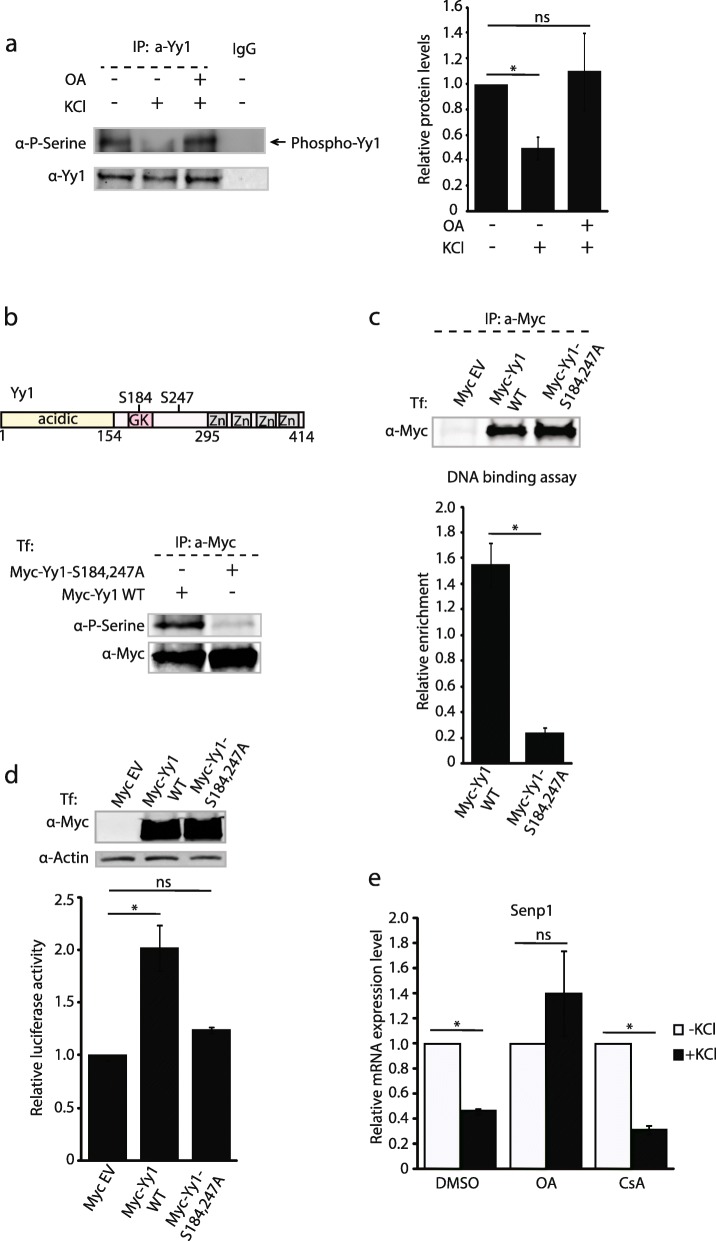
Membrane depolarization mediates a decrease in Yy1 phosphorylation and depletes Yy1 from the *Senp1* promoter via the PP1/PP2A phosphatases. (**a**) Okadaic acid (OA) prevents Yy1 de-phosphorylation upon neuronal depolarization. Left: mouse cortical neurons were treated with or without neuronal depolarization with 60 mM KCl for 2 hrs, or pretreated with 100 nM OA for 4 hrs followed with 2 hr treatment with 60 mM KCl. Endogenous Yy1 was pulled down and Phosphorylated Yy1 was detected with anti-phospho-Serine antibodies. Right: quantification of phosphorylated Yy1 levels versus total Yy1. (**b**) upper panel: a schematic diagram of Yy1 protein and the location of serines S184 and S247 relative to the acidic N-terminus, the glycine-lysine-rich central (GK), and the C-terminal DNA-binding zinc (Zn) finger domains. Lower panel: S184, 247A mutation inhibits Yy1 phosphorylation. (**c**) Inhibition of Yy1 phosphorylation leads to the reduction of its binding to *Senp1* promoter in vitro. Upper panel showed the purified proteins for in vitro binding assay by Western blotting. In vitro binding of Myc-Yy1 wild type (Myc-Yy1-WT) and Myc-Yy1-S184, 247A to the DNA fragments containing *Senp1* promoter was assessed with qRT-PCR. Graphs showed the relative enrichment after normalization with the enrichment in the control (Myc alone). (**d**) Yy1 phosphorylation is necessary for its activation on *Senp1* promoter. Upper panel: Western blot with anti-Myc antibodies showing the expression of Yy1 wild type and mutant in the cells applied for luciferase assay. Lower panel: Luciferase activity was measured in Neuro2A cells co-transfected with *Senp1* promoter fused to the luciferase reporter and Myc alone, Myc-Yy1-WT, or Myc-Yy1-S184, 247A, respectively. The luciferase activity from cells transfected Myc alone was set at 1. (**e**) Effects of various phosphatase inhibitors on KCl induced repression of *Senp1* mRNA. Cortical neurons were pretreated with 100 nM Okadaic acid (OA) for 4 hrs or 50 μg/ml Cyclosporin A (CsA) for 22 hrs followed with 2 hr treatment with 60 mM KCl. *Senp1* expression was detected by qRT-PCR. Graphs indicate three independent biological replicates. Error bars represent one standard deviation from the mean. *(p < 0.05). p value was determined using two-tailed unpaired *t* test.

One way that neurons employ for a rapid modulation of their transcription program in response to neuronal activity is by regulating the post-translational modifications (PTMs) of epistatic transcription factors. Yy1 can be modified by multiple PTMs, such as phosphorylation, acetylation, SUMOylation and ubiquitination [29, 37–42]. These modifications modulate either Yy1’s binding ability to DNA or interaction with protein co-factors [29, 37, 42, 43]. Previous studies have shown that Yy1 phosphorylation modulates its binding to the *Egr2* and *Talin2* promoters, and murine leukaemia virus long terminal repeat [38, 44, 45]. We asked whether the loss of Yy1 binding to *Senp1* promoter upon neuronal depolarization might result from alterations in Yy1 phosphorylation levels induced by neuronal activity. To address this, we immunoprecipitated endogenous Yy1 from resting and depolarized primary neurons and checked its phosphorylation status. Western blot of the immunoprecipitants with anti-phospho-Serine antibodies suggests that neuronal activity reduces Yy1 phosphorylation (Fig. 4a).

During Schwann cell differentiation, Yy1 can be phosphorylated by MEK kinase on serine 184 (S184) and 247 (S247), regulating *Egr2* expression in peripheral nerve myelination [44]. These Yy1 serine residues are conserved in vertebrate species, which underscores their importance. We next tested whether these Yy1 phosphorylation sites could regulate its binding to the *Senp1* promoter. First, these two phosphorylation acceptor sites (S184 and S247) were mutated to alanine, creating the Yy1 mutant (Myc-Yy1-S184, 247A) (Fig. 4b). Yy1 phosphorylation was greatly compromised in the mutated form (Fig. 4b). Both wild type and mutated Myc-tagged Yy1 were immunopurified from Neuro2A cells following transfection and immobilized on the agarose beads (Fig. 4c, top). Their DNA binding abilities to *Senp1* promoter region were tested in vitro [30]. Yy1 occupancy at the *Senp1* promoter was abolished when the phosphorylation acceptor sites were mutated (Fig. 4c, bottom), arguing that Yy1 phosphorylation stabilizes its binding to the DNA of *Senp1* promoter. Consistent with this, the wild type Yy1 activates *Senp1* promoter in a luciferase reporter assay, whereas, the Yy1 mutant lost its ability to activate this reporter (Fig. 4d). Altogether, our results show that upon neuronal activity, the phosphorylation levels of Yy1 are reduced, leading to its deprivation from the *Senp1* promoter and reduced *Senp1* transcription. We identify two serine residues, S184 and S247, within the Yy1 protein that are crucial for the phosphorylation-dependent alterations in *Senp1* transcription.

### The PP1/PP2A phosphatases modulate Yy1 in depolarized neurons

Since Yy1 phosphorylation is critical for binding to the *Senp1* promoter, we asked how membrane depolarization regulated Yy1’s phosphorylation status. Because Yy1 phosphorylation is inhibited in primary cortical neurons upon stimulation by KCl, we speculated that this change might result from the up-regulation of protein phosphatase activities. Neuronal activity enhances protein phosphatases 1 and 2A (PP1/PP2A) and protein phosphatase 2B (PP2B; also known as Calcineurin) [14], so we tested whether the depolarization-induced *Senp1* repression was dependent on these enzymes. Okadaic acid (OA) and cyclosporin-A (CsA) can inhibit the PP1/PP2A and PP2B phophatases, respectively [14]. Primary neurons were treated with these pharmacologic inhibitors before depolarization and their effect on the transcription of *Senp1* was measured by RT-qPCR. Okadaic acid treatment reversed the decrease in *Senp1* levels after depolarization (compare the DMSO vehicle versus OA treatment) (Fig. 4e). In contrast, CsA treatment did not attenuate the repression of *Senp1* caused by neuronal depolarization (Fig. 4e).

We then examined whether inhibition of PP1/PP2A phosphatases enhance Yy1 phosphorylation. Endogenous Yy1 proteins were immunoprecipiated from primary cortical neurons either with or without Okadaic acid treatment. We observed that inhibition of PP1/PP2A phosphatase activity enhances levels of phosphorylated Yy1 (Fig. 4a). Collectively, our results implicate that PP1/PP2A dephosphorylate Yy1 and that their phosphatase activity is indispensable for the neuronal activity-induced *Senp1* deactivation.

### Depletion of Yy1 reduces GluR1 protein expression levels

**Fig. 5:**
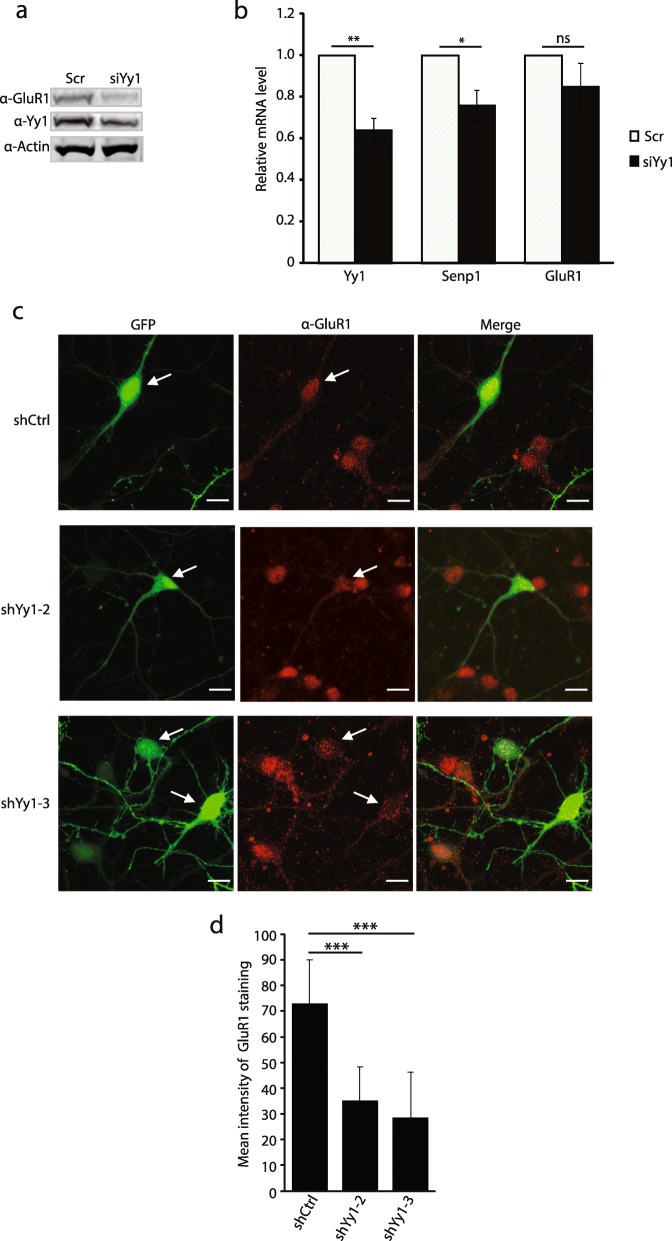
Depletion of Yy1 in primary cortical neurons reduces GluR1 protein levels. (**a**) Western blot from primary neurons after knockdown of Yy1 with siRNA (siYy1). Primary cortical neurons were transfected with scramble (Scr) or siYy1. Whole cell extracts were prepared and subjected to immunoblot with anti-GluR1, anti-Yy1, and anti-Actin antibodies. (**b**) qRT-PCR shows that Yy1 and Senp1 mRNA levels are statistically decreased following siYy1, while *GluR1* mRNA levels are not statistically decreased. Graphs indicate three independent biological replicates. Error bars represent one standard deviation from the mean. **(*p* < 0.01); *(p < 0.05); ns. not significant (*p* > 0.05). p value was determined using two-tailed unpaired *t* test. (**c**) Anti-GluR1 immunostaining of the shRNA transfected cells. Primary cortical neurons were transfected with shRNA Control (shCtrl), shYy1–2, or shYy1–3. GFP encoded in the shRNA vector tracks the transfected cells. Scale bar: 10 μM. (**d**) Quantification of GluR1 level in control and shYy1 transfected neurons. The mean intensity of GluR1 signals was determined using Image J software. *** (*p* < 0.0001). p value was determined using two-tailed unpaired *t* test

Senp1 is important for homeostatic synaptic scaling, controlling the trafficking of AMPA receptors (AMPARs), particularly the GluR1 subunit [20, 21]. The over-expression of Senp1 prevents the increased GluR1 surface levels following glycine-induced AMPAR expression [21]. We asked whether Yy1 could alter the total cellular levels of GluR1. Knockdown of Yy1 depleted the total GluR1 protein levels in primary cortical neurons (Fig. 5a). Interestingly, while *Senp1* transcription is reduced, depletion of Yy1 did not significantly affect the mRNA levels of the *GluR1* (also known as *Gria1*) gene (Fig. 5b), suggesting that Yy1 does not directly regulate *GluR1* transcription. To further support that Yy1 regulates the expression of GluR1 at the protein level, primary cortical neurons were transfected with two different Yy1 shRNAs that have a coupled GFP expression enabling the identification of individual transfected neurons to knockdown Yy1. GluR1 immunostaining was performed on these neurons. Following Yy1 depletion we see a diminution of GluR1 immunostaining (Fig. 5c). The intensity of GluR1 staining was quantified and a statistically significant decrease in GluR1 signals was observed in Yy1 knockdown neurons (Fig. 5d). In addition, surface GluR1 was detected in neurons under non-permeant conditions and we found that Yy1 depletion also reduced the surface expression of GluR1 (Additional file 2: Figure S2). These findings suggest that Yy1 controls the expression of the GluR1 protein in neurons.

### Senp1 overexpression rescues GluR1 levels following Yy1 depletion

**Fig. 6:**
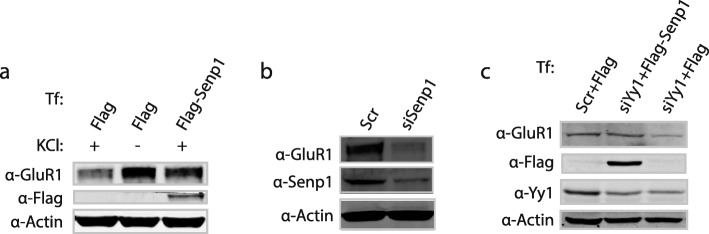
Yy1 modulates Senp1 to promote GluR1 expression. (**a**) Senp1 overexpression overcomes the reduction of GluR1 expression following membrane depolarization. Primary cortical neurons were transfected (Tf) with Flag alone or Flag-Senp1 with or without KCl treatment. Whole cell extracts were prepared and subjected to immunoblot with anti-GluR1, anti-Flag, and anti-Actin antibodies. Actin is used as a loading control. (**b**) Depletion of Senp1 reduces GluR1 protein. Total proteins were extracted and the expression of Senp1 and GluR1 were detected by western blotting. (**c**) Senp1 alleviates the decrease of GluR1 caused by Yy1 depletion. Yy1 siRNA (siYy1) or scramble control (scr) siRNAs together with Flag alone (+Flag) or Flag-Senp1 (+Flag-Senp1) were transfected into cells. Whole cell lysates were prepared 48 h later and subjected to western blot with anti-GluR1, anti-Flag, anti-Yy1, and anti-Actin antibodies. Actin was used as a protein loading control

Because Yy1 does not regulate *GluR1* mRNA, we queried how Yy1 contributes to GluR1 expression in neurons. Previous studies have suggested that Senp1 plays a role in the localization of GluR1. As our above results indicate that Yy1 can regulate Senp1 expression, we questioned whether Senp1 also modulates the total amount of GluR1 in addition to its localization. We first transfected primary cortical neurons with a control or Flag-Senp1 expression plasmids under depolarizing or non-depolarizing conditions. Consistent with previous studies [10–13], neuronal activity reduces GluR1 expression (Fig. 6a). Interestingly, the over-expression of wild type Senp1 under depolarizing conditions rescued the expression of GluR1 (Fig. 6a). In contrast, depletion of Senp1 by siRNA reduced GluR1 expression (Fig. 6b).

Next, we asked whether Senp1 mediated Yy1’s regulation of GluR1 expression. To address this, we depleted Yy1 by siRNA and over-expressed a Flag-tagged full-length Senp1 protein in resting neurons (Fig. 6c). Yy1 knockdown reduced the levels of GluR1 protein while the overexpression of Flag-Senp1 indeed rescued the GluR1 levels (Fig. 6c). The over-expression of Senp1 protein did not affect the total level of endogenous Yy1 (Fig. 6c). Altogether, we determined that Yy1 controls GluR1 protein levels through Senp1 expression.

## Discussion

**Fig. 7:**
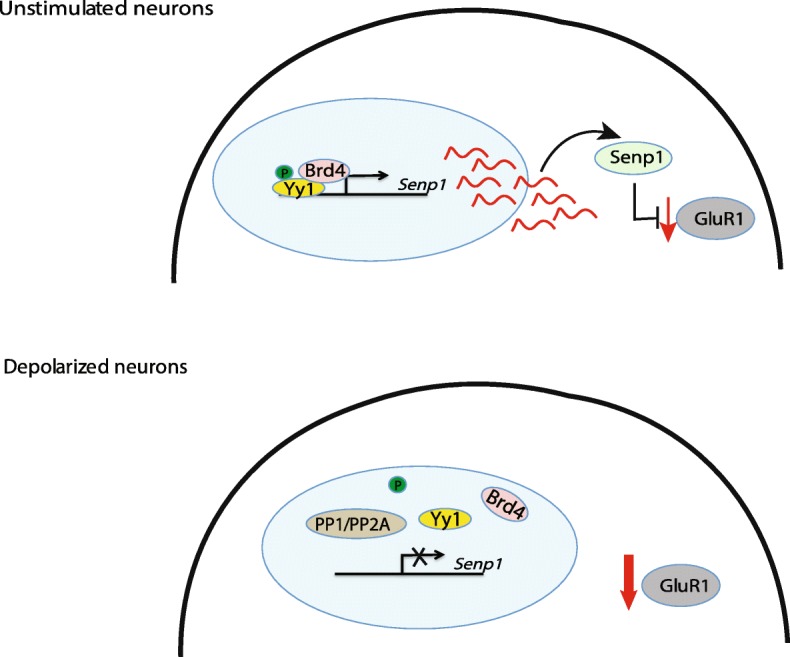
Model for Yy1 modulation in *Senp1* expression and GluR1 protein levels following membrane depolarization. Yy1 is phosphorylated and binds to the *Senp1* promoter where it recruits the BET family member Brd4 and activates *Senp1* transcription in unstimulated neurons. Senp1 protein promotes the expression of GluR1 subunit. Upon neuronal depolarization, PP1/PP2A phosphatase activity is enhanced and dephosphorylates Yy1, resulting in its eviction from the *Senp1* promoter. The Yy1-interacting partner Brd4 is also removed simultaneously. Consequently, the Senp1 protein expression is compromised resulting in a depletion of GluR1

In this study, we show that Yy1 modulates *Senp1* expression in response to membrane depolarization and propose the following model of action (Fig. 7). In non-depolarized neurons, Yy1 is phosphorylated and binds to the *Senp1* promoter where it recruits the BET family member Brd4 and activates *Senp1* transcription. Senp1 protein promotes the expression of GluR1 subunit, a pivotal component of glutamate signaling in learning and memory. Upon neuronal depolarization, PP1/PP2A phosphatase activity is enhanced and dephosphorylates Yy1, resulting in its eviction from the *Senp1* promoter. The Yy1-interacting partner Brd4 is also removed from the *Senp1* promoter during depolarization. Consequently, the Senp1 protein expression is compromised resulting in a depletion of GluR1. Currently, the mechanisms of Senp1 in regulating GluR1 expression is not clear. Previous studies have shown that protein Sumoylation regulates protein stability through its crosstalk with protein Ubiquitination [50]. As one of the major SUMO proteases, Senp1 may be capable of regulating GluR1 Ubiquitination, which is known to be able to control its protein stability [13].

Protein SUMOylation is dynamic during neuronal activity [16], but it is unknown how it is regulated. The ablation of the *Senp1* gene in mice causes embryonic lethality [46], precluding studies to determine its role in mature neurons. Our studies show that neuronal activity represses *Senp1* levels. In addition, recent studies also suggest that the distribution of Senp1 in neurons are regulated by neuronal activity [47]. As a major regulator of protein SUMOylation [18, 29], the repression of *Senp1* results in an increase of SUMOylation in activated neurons. Senp1 regulates homeostasis synaptic scaling by controlling the trafficking of the GluR1 [20, 21]. Our results suggest that both Senp1 and its upstream regulator, Yy1 promote the stability of GluR1 protein expression.

In neurons, protein SUMOylation can directly regulate its substrates activities by altering protein-protein interaction, stability, and localization [16, 18, 46]. Arc SUMOylation modulates its localization and is required for some forms of long-term potentiation consolidation and homeostatic scaling in neurons [20, 48]. For GluK2, SUMOylation is necessary for its trafficking and its functions in synaptic plasticity [49]. Conversely, SUMOylation also influences its targets indirectly by its crosstalk with other PTMs, such as phosphorylation [50, 51]. PTM crosstalk may be crucial in regulating neuronal development and activity [51]. Several proteins such as Creb and Mecp2, both important for neuronal function, can be phosphorylated and SUMOylated [52, 53]. Senp1 regulates calcium influx and glutamate release from presynaptic terminals and eventually contribute to synaptic functions by modulating protein SUMOylation at the synapse [22]. These studies further highlight the importance of the down-regulation of *Senp1* in modulating the dynamics of protein SUMOylation and shaping the neuronal response to any stimuli. Although there are 6 members in Senp family [18], our results suggest that their responses to neuronal activity are as different as their protein substrate specificities [18, 46]. Thus, SUMOylation may play an equally important role as phosphorylation in neuronal activity.

Several studies have demonstrated the importance of Yy1 in neuronal development [43, 44]. Mouse homozygous mutations of *Yy1* are peri-implantation lethal [54]. Loss of one *Yy1* allele in mice results in neurulation defects with a subset of *Yy1* heterozygous mice having exencephaly [54]. The function for Yy1 in mature neurons is not clear although it is highly expressed in these cells [29, 37, 55]. Yy1 regulates the transcription of *MMP9* and *miRNA-190*, which are pivotal for physiological and pathological neuronal plasticity, suggesting that Yy1 may be also important for mature neuron activity [37, 45]. Here we identify another target of Yy1 in neurons, *Senp1*. In addition, we find that the Yy1 co-factor Brd4 positively regulate *Senp1* in resting neurons. Similar to *Yy1*, mice lacking *Brd4* results in peri-implantation lethality [56], suggesting that it plays a crucial role in early development. Consistent with this, our recent study demonstrates that Brd4 controls pluripotent gene regulation [28]. Very little is known, however, about Brd4’s function in neuronal development and in neuronal activity.

Yy1 targets transcription factors, such as Egr2 and c-fos, known regulators of late response genes in neuronal depolarization. This suggests that Yy1 mediated alteration of gene transcription could be an epistatic event during neuronal activity. Therefore, together with previous reports [37, 45, 55, 57], we believe that Yy1 could be a pivotal mediator in neuronal plasticity by regulating the transcription of many genes besides *Senp1* in mature neurons. Because Yy1 can function as either a repressor or an activator dependent on its interaction partners [31], it is very likely that Yy1 is able to bi-directionally monitor the gene transcription in neurons upon neuronal depolarization. This property is quite different from other transcription factors that configure neuronal responses to various stimuli, such as Creb, which always activates gene transcription [16, 58]. Previous studies have shown that Yy1 represses *c-fos*, and activates *Cdk6, Line1,* and *c-Myc* [44, 59–61]. This effect of KCl stimulation could be achieved by the pathway that neuronal activity regulates the phosphorylation of Yy1, which then modulate its binding to DNA. How neuronal activity controls Yy1’s function at these regions and what co-factors Yy1 interacts with will require further studies.

## Conclusions

We present that neuronal depolarization represses *Senp1* transcription by the modulation of Yy1 and recruitment of BET family members. In addition, the expression of Senp1 regulates the level of GluR1 in neurons. AMPARs are pivotal for long-lasting memory, dynamic changes in neuronal synaptic efficiency or plasticity and synapsis at many postsynaptic neurons. Therefore, the modulation of Yy1 and Senp1 on GluR1 may play important roles in mature neuron functions including neuronal plasticity and could be involved in some neuronal degenerative diseases [29].

## Supplementary information

**Additional file 1: Figure S1.** Membrane depolarization represses the expression of *Senp1* and *Tet1.* (a) The relative mRNA levels of *Bdnf IV* in cortical neurons was determined by qRT-PCR following treatment with vehicle (Control), 60 mM KCl for 2 hrs, or 24 hrs. (b) Western blot analysis of phosphorylated Creb level following neuronal depolarization in cortical neurons. Total proteins were extracted from cortical neurons after 2 hr treatment with 60 mM KCl and vehicle. Actin was used as loading control. (c-d) The expression of indicated genes in cortical neurons were determined by qRT-PCR following treatment with vehicle, 60 mM KCl for 2 hrs (c), or 24 hrs (d). Only Senp1 and Tet1 were significantly reduced by neuronal activity. Graphs indicate three independent biological replicates. Error bars represent one standard deviation from the mean. *(*p* < 0.05). *p* value was determined using two-tailed unpaired *t* test. (e) Western blot analysis of Senp1 and Yy1 level after treatment with TTX and KCl in cortical neurons. Total proteins were extracted from cortical neurons after 2 hr treatment with 60 mM KCl, 1 μM TTX and vehicle. Actin was used as loading control.

**Additional file 2: Figure S2.** Depletion of Yy1 reduces surface GluR1 in primary cortical neurons. (a) Immunostaining of surface GluR1 in shRNA transfected cells. Primary cortical neurons were transfected with shRNA Control (shCtrl), shYy1–2, or shYy1–3. GFP included in the shRNA vector tracks the transfected cells. Scale bar: 25 μM. (b) Quantification of surface GluR1 level in control and Yy1 depletion neurons. The mean intensity of GluR1 signals was determined using Image J software. *** (*p* < 0.0001). p value was determined using two-tailed unpaired *t* test.

## Data Availability

All data generated or analyzed during this study are included in this article and its supplementary information files.

## Abbreviations

BET: Bromodomain and extraterminal
Brd4: Bromodomain-containing protein 4
CoIP: Co-immunoprecipitation
CsA: Cyclosporin-A
GluR1: Glutamate receptor GLUR1
OA: Okadaic acid
PP1: Protein phosphatases 1
PP2A: Protein phosphatases 2A
qChIP: Quantitative chromatin immunoprecipitation
RNAP Pol II: RNA polymerase II
Senp1: Sentrin-specific protease 1
SUMO: Small Ubiquitin like Modifier
Yy1: Yin Yang 1
