# Supplementary figures and images for: Yy1 regulates Senp1 contributing to AMPA receptor GluR1 expression following neuronal depolarization
Source: J Biomed Sci. 2019 Oct 20;26:79. doi: 10.1186/s12929-019-0582-1 (PMC6800989; doi:10.1186/s12929-019-0582-1)

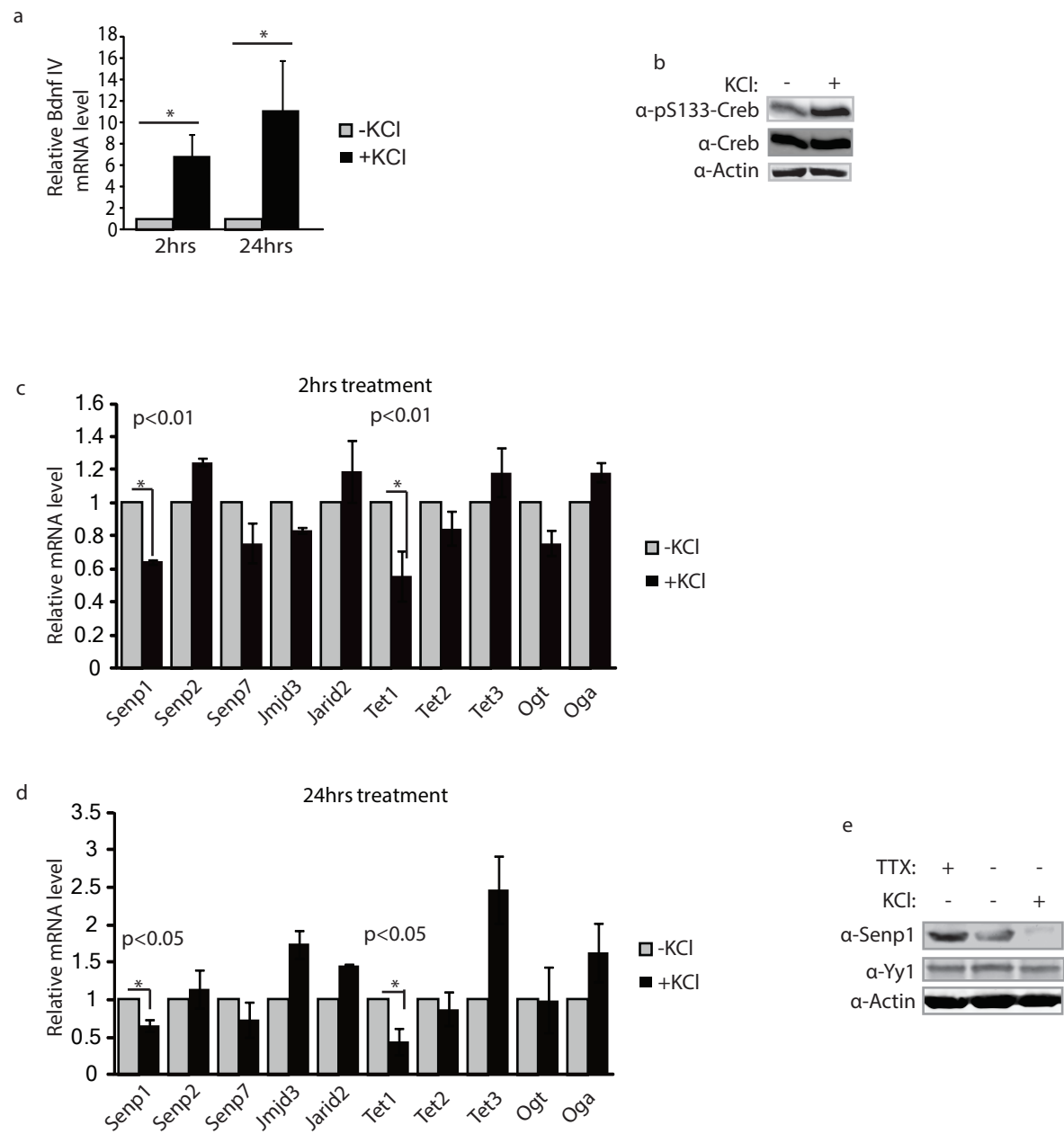

Supplement: Supplementary file 1 — Additional file 1: Figure S1. Membrane depolarization represses the expression of Senp1 and Tet1. (a) The relative mRNA levels of Bdnf IV in cortical neurons was determined by qRT-PCR following treatment with vehicle (Control), 60 mM KCl for 2 hrs, or 24 hrs. (b) Western blot analysis of phosphorylated Creb level following neuronal depolarization in cortical neurons. Total proteins were extracted from cortical neurons after 2 hr treatment with 60 mM KCl and vehicle. Actin was used as loading control. (c-d) The expression of indicated genes in cortical neurons were determined by qRT-PCR following treatment with vehicle, 60 mM KCl for 2 hrs (c), or 24 hrs (d). Only Senp1 and Tet1 were significantly reduced by neuronal activity. Graphs indicate three independent biological replicates. Error bars represent one standard deviation from the mean. *(p < 0.05). p value was determined using two-tailed unpaired t test. (e) Western blot analysis of Senp1 and Yy1 level after treatment with TTX and KCl in cortical neurons. Total proteins were extracted from cortical neurons after 2 hr treatment with 60 mM KCl, 1 μM TTX and vehicle. Actin was used as loading control. [file 12929_2019_582_MOESM1_ESM.pdf]

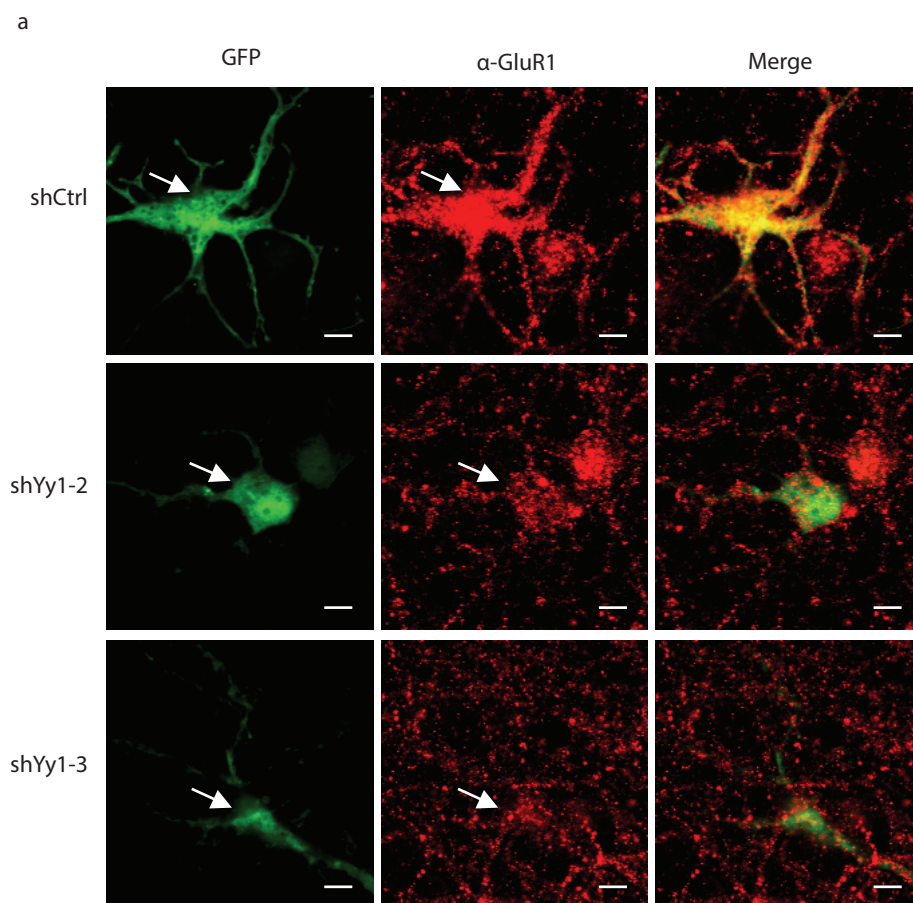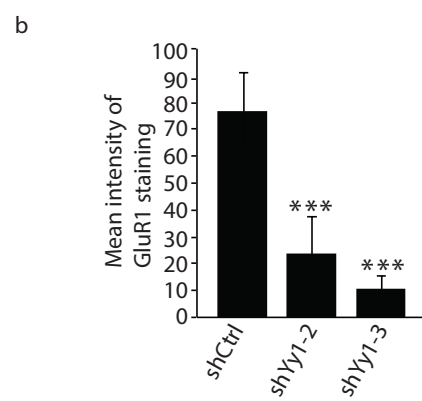

Supplement: Supplementary file 2 — Additional file 2: Figure S2. Depletion of Yy1 reduces surface GluR1 in primary cortical neurons. (a) Immunostaining of surface GluR1 in shRNA transfected cells. Primary cortical neurons were transfected with shRNA Control (shCtrl), shYy1–2, or shYy1–3. GFP included in the shRNA vector tracks the transfected cells. Scale bar: 25 μM. (b) Quantification of surface GluR1 level in control and Yy1 depletion neurons. The mean intensity of GluR1 signals was determined using Image J software. *** (p < 0.0001). p value was determined using two-tailed unpaired t test. [file 12929_2019_582_MOESM2_ESM.pdf]
